# The "Universal Review"

**Published:** 1888-07-28

**Authors:** 


### THE "UNIVERSAL REVIEW."

Decidedly the most popular article in this month's *Universal Review* will be Mr. Samuel Butler's "Quis Desiderio?" It narrates how he was almost driven out of the profession of letters by the disappearance from the reading-room of the British Museum of Frost's "Lives of Eminent Christians," with which he was wont to construct for himself a temporary sloping desk. He could not compose in any other place, and he could not write at a flat table, and despair filled his breast till Professor Garnett restored the volume—removed by some mysterious means "through the posthumus machinations of the late Mr. Darwin"—to its accustomed place. Other reading-room experiences are recorded—for example, the pathetic request of the clerks that Mr. Butler, who rejoices only in a modest B.A. degree, would try for an M.A. in order to evade changes in the catalogue. "I said," Mr. Butler tells us, "that a Mastership was an article the University could not do under about five pounds, and that I was not disposed to go sixpence higher than three ten. They again said it was a pity, for it would be very inconvenient to them if I did not keep to something between a bishop and a poet. I might be anything I liked in reason, provided I showed proper respect for the alphabet; but they had got me between 'Samuel Butler, bishop,' and 'Samuel Butler, poet.' It would be very troublesome to shift me, and bachelor came before bishop. This was reasonable, so I replied that under those circumstances, if they pleased, I thought I would like to be a philosophical writer." The exquisite humour of this paper is a delicious relief to one's mind from the very serious matters discussed in the other articles, most of which make one think deeply and sadly. Among these may be placed Mr. Llewelyn Davies's "Problem of Poverty," which starts with the depressing but practical paradox that "the more you give to the poor the poorer they become;" and George Fleming's analysis of that "Deficiency in Women"—a refusal or incapacity to carry out their thoughts to a just and logical conclusion. The writer shows that women, even when they have opinions, have not the courage of them, so hemmed in are they by conventional limits—those moral pinafores which are worn by all girl children. The very fuss we make about our clever women proves how rare they are. If we had a Miss Ramsay every year we should cease to get excited about her. But George Fleming does not quite go to the root of the matter, which perhaps was stated unconsciously by Milton in his definition of the religious positions of men and women, "He for God only, she for God in him." Man's approval is the goal of woman's ambition; women are what men want them to be, and on the whole men don't—as yet—want women to be too logical. It may be very sad; it may be very stupid; but it is an ineradicable sex-instinct, and while it exists women will continue to be just what men make them. Two of the articles in the *Review* are replies to papers that appeared last month. Mr. T. P. O'Connor, M.P., takes up the cause of "Home Rule and the Opposition Leaders," and Mr. Arthur Smith protests against Mr. Grant Allen's deification of all things Celtic by writing "The Defence of the Teuton." Mr. Quilter, in a second paper on the Salon, preaches with all his usual force and earnestness the doctrine that it requires more than cleverness to make a picture, and that painting is not merely a trick of manipulating colour with a brush, but the expression of a man's soul in form and colour. The moral of Mr. Henry James's "Lesson of the Master," so far as it has gone, seems to be that success is apt to have a deteriorating effect on a writer's literary work.

